# Autocatalytic Selection as a Driver for the Origin of Life

**DOI:** 10.3390/life14050590

**Published:** 2024-05-06

**Authors:** Mike P. Williamson

**Affiliations:** School of Biosciences, University of Sheffield, Sheffield S10 2TN, UK; m.williamson@sheffield.ac.uk

**Keywords:** evolution, origin of life, prebiotic, natural selection, extraterrestrial life

## Abstract

Darwin’s theory of evolution by natural selection was revolutionary because it provided a mechanism by which variation could be selected. This mechanism can only operate on living systems and thus cannot be applied to the origin of life. Here, we propose a viable alternative mechanism for prebiotic systems: autocatalytic selection, in which molecules catalyze reactions and processes that lead to increases in their concentration. Crucially, this provides a driver for increases in concentrations of molecules to a level that permits prebiotic metabolism. We show how this can produce high levels of amino acids, sugar phosphates, nucleotides and lipids and then lead on to polymers. Our outline is supported by a set of guidelines to support the identification of the most likely prebiotic routes. Most of the steps in this pathway are already supported by experimental results. These proposals generate a coherent and viable set of pathways that run from established Hadean geochemistry to the beginning of life.

## 1. Introduction

How did life begin on Earth? We have made good progress towards answering this question. Once something like a cell exists (with some kind of genetic material, macromolecular catalysts, and a cell membrane), then a broad outline of a pathway is clear. There are also plausible suggestions as to how the conditions on an early Hadean Earth could have produced usable concentrations of tricarboxylate cycle (TCA) intermediates. However, there is a large gap between these two and no clear way of identifying how to bridge it. Two leading researchers have commented, “Origin of life studies is a field with an extraordinary diversity of proposals, but few good ways to systematically judge the framing of questions and the prioritization of evidence” [1]. In other words, we do not know how to ask good questions, or how to recognize good answers [2]. Here, we propose a set of guidelines (see also [3,4]), and use these to outline a plausible scenario, relying heavily on the novel mechanism of *autocatalytic selection.* We use these guidelines to propose routes leading from simple starting materials to sugar phosphates, lipids, amino acids, nucleotides, and their polymers (in particular, proteins). In each case, autocatalytic selection suggests a different original function for these metabolites than those that are now more familiar to us, which supports a buildup in their concentrations.

Below, we set out the guidelines and then suggest possible mechanisms for autocatalytic selection of the major classes of metabolites. However first, we outline a route that could have generated TCA intermediates on an early Earth based on alkaline thermal vents. This is only one of several possibilities (some others being panspermia [5], Urey/Miller-type environments [6,7,8,9], and various subaerial environments such as Darwin’s classic “warm little pond”, as well as other thermal vents such as the more familiar black smokers) but serves to introduce some important concepts and to bring us to a suitable starting point.

The early Hadean Earth is widely supposed to have been warm and reducing, with very low amounts of free oxygen [1,3,10]. There must have been frequent meteorite impacts, which may well have brought a wide range of organic precursors, as well as water. However, the general view is that for life to begin, the environment should be fairly stable over long periods of time to allow pre-metabolite concentrations to build up gradually. It has been cogently argued that in order to drive suitable prebiotic chemistry, it is necessary to have a continuous energy source [11]. One such energy source is an alkaline hydrothermal vent [12,13,14,15,16]. These are undersea regions where water seeps into the Earth’s crust and reacts with the hot rocks there, including the mineral olivine (roughly Mg_1.6_Fe_0.4_SiO_4_), to produce hydrogen and CO_2_ [17], which react together to produce methane plus other hydrocarbons and CO, in a process called serpentinization (after the mineral serpentine, also produced in this reaction) [18,19]. These vents are warm rather than hot (40–90 °C), which is consistent with the genetic evidence on the origins of life [20,21]. The emerging gases have a wide range of pH, but where they emerge away from the crustal ridge (such as the region known as the Lost City in the mid-Atlantic [18]), they are alkaline with a pH of 9–11. The significance of this process is that it produces large volumes of gas and has been running continuously probably for millions of years [22,23,24], thus providing an environment that is stable (although far from equilibrium) over very long periods of time. These vents contain rocks rich in Fe^2+^, Ni^2+^ and other metals such as molybdenum and sulfides, and are good heterogeneous catalysts for a wide variety of reactions [25]. A further feature of these alkaline vents (as seen now and presumably earlier also) is that the chimneys that are produced contain very large amounts of microscopic compartments. They thus present a large surface area (suitable for surface catalysis) and are a good environment for trapping and retaining chemicals [26]. It has been pointed out that one of the most phylogenetically widespread enzymes, and therefore an enzyme likely to have been present in the earliest living organisms, is ferredoxin, which contains an Fe,S cluster [27]. This supports the idea of Fe(Ni)S catalysts as important for prebiotic chemistry. A related argument is the observation that many of the cofactors for the most deeply rooted biosynthetic pathways use cofactors such as pterins, which have metal thiols at their centers, further strengthening the case for metal thiols as catalysts for early chemistry [28].

A leading proponent of the iron sulfide scenario was Wächtershäuser, who developed extensive experimental evidence that iron sulfides could catalyze important prebiotic reactions [4,29]. He also noted that all of the molecules thought to be fundamental to prebiotic chemistry (components of the tricarboxylic acid cycle, acetate, thioacetate, and sugar phosphates) are negatively charged at neutral or alkaline pH and proposed that this would be useful for keeping them close to the positively charged metal sulfide surface, therefore preventing them from diffusing away [8,29]. Green rust (a mixed iron oxide) may also have played a similar role [30,31].

Serpentinization fixes CO_2_ into a range of small organic molecules like methane, acetic acid and thioacetic acid. It has been pointed out that in modern organisms, there are six routes for carbon fixation, of which the simplest is the Wood–Ljungdahl pathway, which is well established as a primitive pathway [20,32]; the chemistry involved in this pathway is similar to serpentinization, supporting the idea that this may have been a major route for carbon fixation under Hadean conditions [26,33].

**Figure 1:**
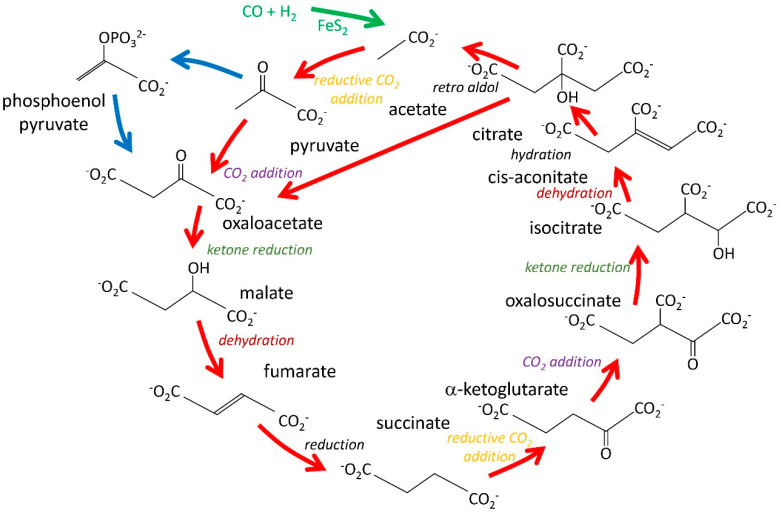
A possible prebiotic reductive TCA cycle. The acids shown here differ in some places from those in a standard TCA diagram and are drawn such that diametrically opposite acids and the reactions that convert them are homologous. Homologous reaction types are indicated in different colored italic fonts, while the three reactions in black font are not homologous. The figure also indicates possible input to the cycle from CO and hydrogen catalyzed by iron pyrite (green) and an alternative route from pyruvate to oxaloacetate that goes via phosphoenolpyruvate (blue arrows), which functions to link the TCA cycle to pathways involving phosphosugars. Many of these compounds are the starting points for important modern biosynthetic pathways, supporting their inclusion in an early autocatalytic cycle: α-ketoglutarate for amino acids (and via glutamine for the nitrogen in all amino acids); acetate for fatty acids and isoprenoids (i.e., all lipids in all three branches of life); pyruvate for sugars and alanine; oxaloacetate for aspartate and pyrimidines; and succinate for pyrroles. We note that the steps from oxaloacetate to succinate are chemically identical to the modern route for fatty acid synthesis. Diagram based on Figure 4.1 in [1]. There is also a similar diagram in [28].

Following the initial fixation of carbon, one next needs reactions that incorporate the carbon into a range of metabolites. It has been argued that the best way to do this is via one or more autocatalytic cycles: a cyclic series of reactions, in which one turn around the cycle regenerates the original members, with an increased amount of one member [34]. In this way, repeated turns around the cycle lead to increases in the concentrations of all the members of the cycle. This will only happen if leakage from the cycle via side reactions does not drain away metabolites. In order to avoid such leakage, one requires (a) continual input of energy to drive the reactions around the cycle, which is provided by the continuous serpentinization process, and (b) side reactions should be limited in extent, or should give products that are in equilibrium with cycle members. The most widely discussed such autocatalytic cycle is the tricarboxylic acid cycle (TCA cycle, also known as the Krebs cycle) [1]. In present-day aerobic or microaerobic organisms, this is generally an oxidative process, liberating two CO_2_ on every turn around the cycle and generating energy. However, originally, in a reductive environment, it would have run in the reductive direction. This requires energy input (not a problem close to a hydrothermal vent) and incorporates CO_2_ to build new metabolites: it is an autocatalytic cycle, continually being fed precursors by the serpentinization process (Figure 1) [35].

This reductive TCA (rTCA) cycle has a number of useful features. Many of the reactions are close to equilibrium and so can run backward as well as forward, catalyzed by Fe/S [36]. The cycle, as usually described, has internal duplication of reaction types, as indicated by the colors in Figure 1, meaning that it would require relatively few types of catalytic mechanisms to help drive it. Most of the reactions indicated in Figure 1 have been shown to be possible under potential prebiotic conditions [28,37].

The rTCA autocatalytic cycle can produce high concentrations of the metabolites shown in Figure 1. However, it is much less clear how the other metabolites necessary for life could have appeared. Below, we set out four guidelines and then show how the fourth of these (autocatalytic selection) provides a mechanism for the production of metabolites.

## 2. Guidelines

### 2.1. Guideline 1: Specificity Increases over Time

The earliest chemical systems must have lacked specificity: they will have produced multiple products, lacked control, and only acquired specificity (for example, catalysts to produce specific products) very gradually. This is far from being a new proposal [38] but has some important implications:

1. Early synthesis of a polymer with a defined sequence is so specific as to be highly unlikely. This rules out any scenario in which sequence-specific RNA-like polymers control reaction pathways [28,39,40].
2. More specifically, it implies that a primitive genetic code (that is, a relationship between specific nucleotide groups and specific amino acids [41,42,43,44]) must have emerged before the existence of genes and thus that the genetic code was determined largely by chemistry, well before the requirement for genes.
3. In the early stages, we should not expect to find pathways that exist in order to produce individual compounds (for example, a pathway leading specifically to glucose or ribose). Rather, we expect nonspecific reaction types that generate a common pool of equilibrating metabolites (e.g., a set of reactions that produce a collection of sugars in equilibrium). Any pathway that *requires* a specific reaction or product is inherently unlikely.

### 2.2. Guideline 2: Reactions That Resemble Current Biochemical Reactions Are Inherently More Likely

There is no law that says that current enzyme-catalyzed reactions must resemble the earliest prebiotic reactions. However, enzymes act by increasing the rate of the nonenzymic reaction, which means that enzymes generally are recruited and evolve to increase the rate of a nonenzymic reaction [28,38]. This implies that in most cases, enzymes perform similar chemistry as in nonenzymic reactions, in turn implying that the early prebiotic reactions will not be radically different from the current reactions [45], though they are of course likely to be less specific [46]. Some authors have described this as a parsimonious approach, i.e., that we should not consider reactions that are dramatically different from the current ones without very good reason [14,35,47]. It also implies that photosynthesis is unlikely to have been important in the early stages of prebiosis [48].

### 2.3. Guideline 3: No Teleology

This is a fundamental tenet of any kind of evolutionary scheme (except creation science) and requires every development to yield an improvement on what was there before, or at least not a decrease in fitness. We cannot anticipate future benefits. Therefore, for example, amino acids are important current metabolites because they form the monomers for proteins. However, a prebiotic environment cannot anticipate the production of proteins, implying that there must have been some other different need for amino acids. This is one of the key chicken-and-egg problems for origin of life research: how and why would a prebiotic environment produce significant amounts of amino acids or nucleotides if there was no current use for them?

### 2.4. Guideline 4: Autocatalytic Selection

This is the mechanism to solve the problem posed in Guideline 3, and is the only one of these guidelines that contains significant novelty. In a prebiotic environment, there can be no Darwinian natural selection, because there is no competition. To replace natural selection, we propose autocatalytic selection: a chemical entity will increase in concentration if it acts as a catalyst for a reaction that (directly or indirectly) produces more of it [49]. Once its concentration has built up, it can be used for something completely different. This type of change in function is very common in evolution; for example, the ear probably developed as a modification of fish gills [50], and bacterial flagella started as protein-export systems and then developed into adhesion devices before becoming a means of propulsion [51]. Below, we propose that amino acids, nucleotides and lipids all increased in concentration initially not for the functions that they currently have, but for other reasons, and then became co-opted into their current functions later, once their concentrations had become sufficiently high to be useful.

These four guidelines have directed the proposals below.

## 3. Proposals for Autocatalytic Origins of Key Metabolites

### 3.1. Phosphosugars and the Pentose Phosphate Pathway

The challenge set out in the Introduction is that hydrothermal vents and rTCA together can credibly produce useful concentrations (in the mM range) of TCA intermediates. However, in order for biochemistry to begin, we also need amino acids, nucleotides, sugars and lipids. Where do they come from? Our proposals begin with sugars and specifically phosphosugars.

As noted above, alkaline hydrothermal vents generate H_2_ and CO_2_ in large quantities, which go on to produce methane, methanol, CO, CH_3_SH [52], and then CH_3_COSH, an activated form of acetic acid, which can feed into the rTCA [53]. As shown in Figure 1, the rTCA produces pyruvate, which can be converted under prebiotic conditions to phosphoenolpyruvate (PEP). PEP is a gateway into phosphosugars and the pentose phosphate pathway (PPP). The addition of phosphate to sugars requires an available source of phosphate. In modern environments, this is difficult, but recent evidence suggests that phosphates may have been available at much higher concentrations in Hadean conditions [54,55,56]. Phosphorylation is not an easy reaction. There have been several proposals of how phosphorylation could have taken place in prebiotic conditions, including polyphosphates [57] and surface catalysis in aqueous microdroplets [58].

The reactions of the PPP are mainly aldol and retro-aldol reactions, which take place at reasonable rates even uncatalyzed [59]. Some of the reactions are redox reactions (oxidative when the PPP runs in its standard direction, or reductive when it runs in the reverse direction), which can be catalyzed by iron sulfides [60]. Thus, a reductive PPP (rPPP) has been proposed as occurring in prebiotic conditions [4,35]. In keeping with Guideline 1, the rPPP should not be seen as a pathway for producing glucose phosphate, but as a set of reactions for interconverting phosphosugars and for fixing carbon by reductive additions. It could become cyclic by adding a few simple aldol reactions, in which case it would be an autocatalytic cycle that uses reducing power and CO_2_ to generate increasing concentrations of sugar phosphates.

All the sugars in the PPP are phosphorylated, which is important because phosphosugars are much less prone to degradation and side reactions than unphosphorylated sugars [8,59,61,62], are easier to synthesize in a Hadean environment [63], and are negatively charged and thus adhere to a positively charged surface catalyst such as iron pyrite [8]. Morowitz has pointed out that all sugars in intermediary metabolism are phosphorylated, further emphasizing the importance of phosphorylated sugars [64]. The scenario outlined above implies an important role for positively charged metal sulfide surfaces as catalysts, increasing the importance of negatively charged metabolites.

The rTCA and rPPP both require a similar set of conditions and energy sources, and thus can easily co-exist. They are interconnected via phosphoenolpyruvate (Figure 1). This interconnection is beneficial for a prebiotic environment because it creates a larger pool of pre-metabolites and provides a salvage mechanism for side products. Everything in PPP is phosphorylated, and everything in the standard rTCA is not. However, parallel reactions could use either [1].

Because rTCA and rPPP can both form autocatalytic cycles, it is not unreasonable that the concentrations of their constituents could increase gradually to mM levels, driven by serpentinization. As our first application of the guideline of autocatalytic selection, we propose that the environments in which phosphosugars were produced were able to generate even higher concentrations of metabolites (compared to environments that did not have so much phosphosugar) because the phosphosugars “catalyzed” further increases in rTCA and rPPP metabolites and thus led to the selection of these environments. Specifically, we propose that phosphosugars could have provided a credible precellular coat. Phosphosugars are negatively charged. They therefore bind to positively charged surfaces such as iron pyrite. Phosphosugars can be chelated by metal ions [65] and stabilized by intermolecular hydrogen bonding. The negatively charged aggregates would adhere to the iron pyrite surface, repel the negatively charged metabolites of the TCA and PPP trapped inside the coat, and hinder their loss by diffusion. Such a function would lead to autocatalytic selection because a coat would permit the buildup of greater concentrations of pre-metabolites inside the coat. These environments are thus “selected for” not by any Darwinian process but because the chemistry allows them to retain higher concentrations of metabolites, leading in turn to higher concentrations of phosphosugars.

Modern-day cells are surrounded by a lipid membrane coat. This membrane is not made of fatty acids in all species; in archaea, the cell membrane is composed of isoprenes, which have a completely different chemical origin. However, in all three kingdoms of life, the membrane is built on a platform of glycerol phosphate, a reduced sugar phosphate. Thus, the common feature of all cell membranes is not fatty acids, but glycerol phosphate, indicating the early importance of glycerol phosphate as a barrier to diffusion. In bacteria, the cell membrane is surrounded by a peptidoglycan wall, which is typically decorated by an extensive array of sugar phosphates, such as the capsule, teichoic acids and lipopolysaccharides. We suggest that this layer preceded the lipid membrane; that is, the original cell envelope was made of sugar phosphates and not of lipids. This proposal removes some of the key difficulties of lipid membrane biogenesis (discussed below), in particular by being less impermeable than lipid membranes. In this context, it is worth noting that glycerol phosphate is not only the common core of all membranes but also the repeating backbone of wall teichoic acids in bacteria like *Bacillus subtilis* and thus an important constituent of two different bacterial coats [66].

When sugar phosphates polymerize, they become even better coats. Such polymers would be abundant, highly negative, and stickier than monomers and therefore good at limiting diffusion, particularly of negatively charged pre-metabolites, which we argue above made up the vast majority of early prebiotic metabolites. They would thus be effective semi-permeable barriers, allowing entry to neutral molecules, such as CO_2_ and hydrogen, and exit to (mostly unwanted) non-negatively charged chemical products but limiting the exit of negatively charged products. There has been much debate over how condensation reactions could have occurred in aqueous solution, and several solutions have been proposed, such as wet/dry cycles [3,67]. We propose another below. Another possible route could be via polyphosphates, which could make condensation reactions more energetically feasible [57]. We note later that metabolism will require the polymerization of amino acids and nucleotides; it is not unreasonable to propose that the conditions that make sugar polymerization possible can be adapted later to support the polymerization of amino acids and nucleotides.

### 3.2. The Autocatalytic Origins of Lipid Membranes

Lipid membranes are a big problem in any discussion of the origins of life. A lipid membrane is an excellent barrier to stop cell contents from dispersing and to prevent the mixing of cell contents with the extracellular medium. It is also fundamental to all current life because transmembrane gradients provide the energy to drive metabolism. However, it also blocks the entry of nutrients and the exit of waste. To allow this, in modern organisms, there are channels, which are complex polypeptides and must therefore have arisen much later than membranes (Guideline 1). This therefore makes an early adoption of lipid membranes inherently unlikely (Guideline 3). Alternatively, the lipid membrane could have been leaky, but a leaky lipid membrane provides little obvious advantage to a proto-cell. How, then, can lipid membranes arise in gradual steps?

We have proposed above that sugar phosphates could have acted as barriers and removed the need for early lipid membranes. However, fatty acids are likely to have been produced in the Hadean environment because the chemistry needed to produce a fatty acid from a β-keto acid is virtually identical to that needed to go from oxaloacetate to succinate in the rTCA (Figure 1) and is thus a likely prebiotic process. Hydrocarbon chains, as seen in fatty acids, are an “over-reduced” metabolic product—too highly reduced to be of much metabolic use to a Hadean economy, where there is no oxygen to oxidize them. We therefore propose that hydrocarbons may have accumulated as waste products of reductive prebiotic chemistry and then gradually been co-opted as platforms for the storage and delivery of other metabolic products. The biochemistry of lipid II is an interesting modern example (Guideline 2) [68]. Lipid II is the precursor for the formation of bacterial peptidoglycan cell walls. It is made of a disaccharide diphosphate, to which is attached an isoprenoid lipid and a peptide. Lipid II is synthesized intracellularly, inserted into the cell membrane, and then flipped to face out of the cell, after which the disaccharide is linked to newly synthesized peptidoglycan to build it up further, the peptide is crosslinked, and the lipid is removed. This unusual process is surprising—unless one sees the lipid anchoring as a way to “park” precursor molecules close together in a hydrocarbon platform. The close proximity of disaccharide precursors raises their effective local concentration and makes it quicker and more favorable to link them together. We thus propose that lipids were adopted as a device for assembling monomeric precursors for the cell wall that were anchored to micelles or vesicles, grew in concentration by autocatalytic selection, and then gradually became useful as membrane components. Their association with sugar phosphates in cell walls would provide a driver for their later adoption as part of a cellular barrier. Unicellular organisms and plants have retained the external polysaccharide cell coat, while for multicellular organisms, we suggest that it proved a hindrance and was therefore discarded. In summary, we propose that lipids accumulated because they increased the rate of polymerization of sugars and thus strengthened the cell wall. Their adoption as membrane components came much later.

### 3.3. The Autocatalytic Origins of Amino Acids

The compounds discussed so far contain C, H, O and P (and probably S too in the form of thioesters) but no N. In order to get anywhere close to modern biochemistry, we need to add N to make amino acids and nucleotide bases; in other words, we need to fix nitrogen. Several possible fixation routes have been proposed [69,70]. Once we have a source of nitrogen, amino acids can be made by transaminations of available ketoacids by ammonia, catalyzed by FeS or Fe(OH)_2_ [70,71].

**Figure 2:**
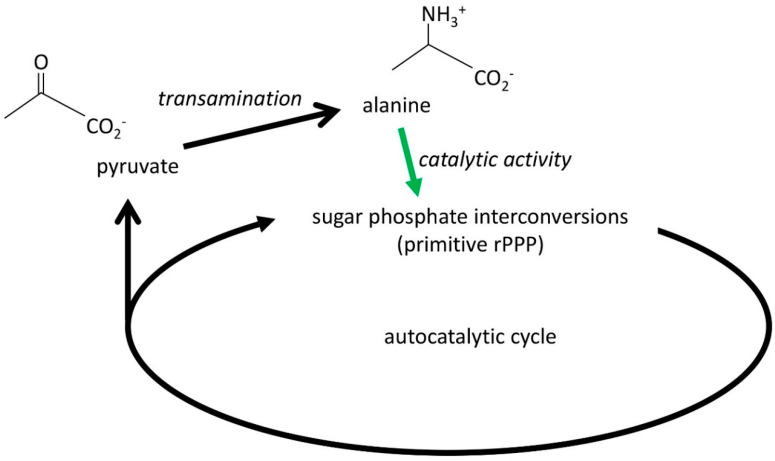
Autocatalytic selection of the amino acid alanine. The autocatalytic cycle for the synthesis of sugar phosphates (a simplified version of the pentose phosphate pathway, running in the reductive direction) generates a range of products, one of which is pyruvate. We propose a reaction that transaminates pyruvate to produce alanine; the alanine then acts as a catalyst to increase the rate and specificity of the sugar-phosphate cycle (green arrow) and thus leads to the production of more alanine because the supply of pyruvate is increased.

However, the key question is, why would a Hadean environment accumulate amino acids if they are not immediately useful? We propose that amino acids were useful initially because they were catalysts. Specifically, as shown by 2021 Nobel prizewinning chemists List and MacMillan [72,73] and others [74,75], they catalyze the synthesis of sugars and deoxysugars [76]. We suggest that if a prebiotic community had the appropriate environmental conditions to produce small amounts of amino acids by transamination from TCA/sugar precursors, then those amino acids would have catalyzed more specific sugar syntheses with fewer side reactions, and thus the autocatalytic accumulation of sugars was faster in that environment. These sugars provided more precursors for amino acid synthesis, and thus an increase in amino acid concentration was autocatalytic. These environments were selected not by competition but because the concentrations of precursors were higher. The increase in amino acid concentration was driven by the energy flow that produces sugar turnover and growth (primarily rPPP); it became gradually more specific in its chemistry, it was autocatalytic, and the amino acids produced remained close to their catalytic origins because of their negative charges (Figure 2).

In passing, we note that iron pyrite crystals are chiral. It is conceivable that the synthesis of amino acids on a chiral surface could produce chiral amino acids [77], which would then catalyze the production of chiral sugars and form the source of chirality in biology [78]. Once a chiral excess has formed (by chance?), it may be promoted because homochiral oligomers appear to be better catalysts than ones of mixed chirality [79].

### 3.4. The Autocatalytic Origins of Nucleic Acids

In origin of life research, we suggest that a nucleotide is best seen as fundamentally a sugar phosphate, with a base attached. In other words, the base is an “add-on” to existing sugar phosphates, with interesting chemical functionality, rather than a fundamentally new category of biomolecule. It is not a credible piece of genetic information without extensive cellular machinery, creating a chicken-and-egg problem over its origins. The ideas presented above, in particular autocatalytic selection, imply that nucleic acids could not have been selected because of their ability to base pair; they must have had (and possibly therefore still have) alternative functions. We can obtain clues about what these may be from a number of sources.

Firstly, Guideline 1 implies that the original functions of nucleotide bases must have been fairly non-specific. In other words, they would have been useful to a prebiotic economy not for any very specific chemical property (i.e., something that adenine can do but guanine cannot) but more for general properties shared by many bases. There are three obvious such properties. They are bases (i.e., they are good at removing acidic protons)—which is useful but, in an alkaline environment, probably not critical because there are other bases present; they are nucleophiles; and some of them are good leaving groups. This third property is interesting and is explored in more detail below. Specifically when compared to primary and secondary amines, they are less basic, they are unable to form Schiff bases (blocking off a range of interesting but unwanted reactions), and they are much better as leaving groups, especially at high pH. This implies that they are able to act as nucleophilic catalysts, in a way not open to primary or secondary amines. We, therefore, propose that the driver for increases in the concentration of nucleotide bases and their analogs was that they were the best available nucleophilic catalysts [15].

Secondly, for similar reasons, the class of “nucleic acid bases” should be expanded to include chemicals with similar composition, structure, function and biosynthesis to the canonical adenine, guanine, thymine, cytosine and uracil. Therefore, we should also include, for example, nicotinamide and pterins [80]. We note that this class, therefore, includes the majority of the enzyme cofactors, further strengthening the case that the reason for their existence was catalysis. In turn, this implies that the concentrations of these compounds must have reached high levels in prebiotic conditions.

**Figure 3:**
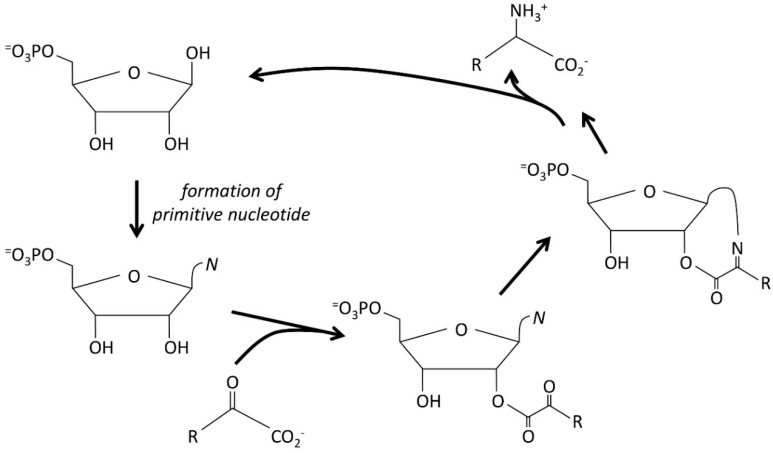
Possible mechanism for the origin of nucleotides. A phosphosugar (ribose is shown here, but any sugar would be possible) has an amine function attached at the anomeric position (*N*). This could be as simple as an amine but will eventually be elaborated to make a nucleotide base (e.g., adenine). It reacts with a ketoacid (from the rTCA) to make an ester. The amine then carries out an intramolecular reaction to make a Schiff base. In a series of reactions, this is reduced and hydrolyzed to make an amino acid and regenerate the phosphosugar. This is an autocatalytic cycle; it catalyzes the transamination reaction to make the amino acid from the ketoacid, which will then go on to be a catalyst for the synthesis of more sugar phosphates (Figure 2) and, therefore, enables more sugar amines to be produced.

Thirdly, the conclusion so far is that there must have been a supply of available nitrogen and that this must have allowed the synthesis of a wide range of nitrogenous bases. Guideline 4 (autocatalytic selection) suggests that the concentrations of these bases increased because they were useful catalysts, but for what? There has been an interesting suggestion that nitrogenous bases might act as catalysts for the formation of more amino acids—most obviously by forming a Schiff base with a ketoacid and, thus (in a reductive environment), eventually leading to a primitive transamination (Figure 3) [41]. This is an attractive idea, not least in that it provides physical interactions between specific bases and specific amino acids and thus points towards a possible origin of the genetic code [41]. This is, therefore, an idea worth considering carefully, but it suffers from the major disadvantage that no reactions like this occur in living systems.

**Figure 4:**
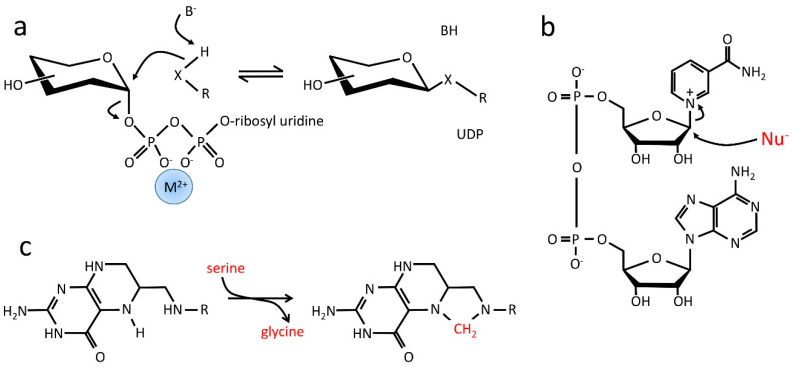
Some functions of nitrogenous bases and related compounds. (**a**) UDP-glucose consists of glucose attached to diphosphoribosyl uridine. It is the substrate for a range of reactions in which glucose is attached to nucleophiles (where X is usually O but could also be N or S). Base-catalyzed nucleophilic attack of the nucleophile leads to displacement of the nucleotide, which forms a good leaving group thanks to the divalent coordinating metal (typically Mg^2+^ or Mn^2+^). Other substrates can also be used—frequently aminosugars or even phospholipids. This reaction is the major route to polymeric sugars. The S_N_2 mechanism shown here is used by GT-A fold enzymes; the GT-B fold enzymes use a different mechanism in which the nucleotide probably leaves prior to the nucleophilic attack, further emphasizing the need for a good leaving group. (**b**) NAD^+^ (nicotinamide adenine dinucleotide) is the substrate for poly(ADP-ribose) transferase (PARP). Nucleophiles attack at the anomeric position, displacing the leaving pyridine ring. (**c**) Tetrahydrofolate acts as a 1-carbon carrier, by coordinating the carbon using a pair of nitrogens. In all of these examples, the base is a carrier, sometimes being directly involved in the chemistry, and sometimes only as a handle. We therefore propose that this was the original function of the nitrogenous bases—to catalyze biosynthetic reactions and thereby stimulate autocatalytic selection [84]. The most interesting of these reactions are those exemplified by UDP-glucose and PARP, where the base is a catalyst promoting the polymerization of a (phospho)sugar. As noted above, this is likely to have been important in the formation of viable cell walls and potentially in the biosynthesis of nucleic acid polymers. Such roles are clearly autocatalytic.

It is, therefore, sensible to ask what current reactions involve nitrogenous bases. The results are interesting:

- The vast majority of current reactions that involve molecules with nitrogenous bases do not actually involve the base in the reaction at all. For example, in the reactions in which ATP or GTP is a substrate for phosphoryl transfer, the base is not involved—the entire phosphoribosyl nucleotide is an elaborate leaving group to facilitate phosphoryl transfer, with the remaining diphosphate coordinated to Mg^2+^ to make it a better leaving group. For reactions involving NAD(P) or FAD in redox reactions, again, the base is not involved in the reaction. In all these cases, the base is used as a “handle” rather than a reactant.
- UDP-glucose acts as a carrier for glucose (Figure 4a); the Mg^2+^:diphosphoribosyl nucleotide is simply a leaving group [81]. In a similar way, NAD is a substrate for reactions involving poly(ADP-ribosyl) transferases (PARPs), where the nicotinamide ring is a leaving group facilitating the transfer of phosphoadenosylribose to a range of nucleophiles (Figure 4b) [82]. Significantly, in both these reactions, the product is a polymer; the function of the nucleotide diphosphate is to “deliver” a new monomer.
- In folic acid derivatives such as tetrahydrofolate (Figure 4c), the nitrogens act as ligands to bind and release a carbon atom and therefore function as carriers for 1C moieties in a range of oxidation states; they are able to act both as nucleophiles and as leaving groups (i.e., as nucleophilic catalysts).
- There are many nitrogenous compounds, such as NAD(P), FAD and molybdopterins, where the nitrogen-containing ring acts as a cofactor for redox reactions (equivalently, it is a carrier for hydride ions) [83].

We noted above that the ubiquity of nitrogenous bases and, in particular, their common use as a handle rather than directly as a reactant implies that they must have been present at high concentrations. We therefore require a simple synthetic route. In modern organisms, the synthesis of nucleotide bases and related compounds is remarkably complicated, i.e., much more elaborate and specific than routes for sugars or amino acids, using aspartate, glutamate, glutamine, glycine, dihydroxyacetone phosphate and phosphoribosyl pyrophosphate as precursors [28,63]. Some also use tryptophan, although because tryptophan is suggested to be a very late arrival to the canonical set of 20 amino acids [85], this is unlikely to have been the original starting material. It is relevant to note that the major route for the biosynthesis of most bases is via salvage pathways where possible. Guideline 2 suggests that current biosynthetic routes are therefore late replacements for a different original pathway.

The bases each contain several nitrogen atoms, implying the need for a plentiful supply of nitrogen at their origin. As noted above, there is so far no convincing long-term source of nitrogen identified for prebiotic development. An interesting suggestion is that the Hadean earth may have had much more intense UV radiation from the young sun than now, which may have powered the conversion of atmospheric N_2_ to a range of nitrogen oxides—a route that is no longer viable because of a reduction in UV intensity [86]. Volcanic lightning could have also contributed significant amounts of nitrogen oxides [87]. There are, however, still no obvious routes from such nitrogen oxides through to bases (which in general have a low oxygen content and therefore do not look like products of nitrogen oxides).

As an alternative, several elegant chemical routes to bases have been proposed that start from hydrogen cyanide (HCN) [61,88,89]. The problem with this idea is that HCN is present now in very low concentrations, and (possibly as a consequence) there are virtually no extant biochemical pathways that use HCN. This does not rule out such pathways–after all, we have just suggested that nitrogen fixation may have occurred originally by a route that is no longer used—but it certainly makes them less likely proposals (Guideline 2) [59,90]. However, it is possible that the Hadean earth had much higher concentrations of hydrogen cyanide than anything possible now, maybe because of meteorite impacts [91]. It has even been suggested that HCN could have kickstarted rTCA metabolism [92]. However, plausible alternative routes have been suggested in alkaline vents that only require ammonia as a nitrogen source [15,90]. The remarkably complicated biosynthetic pathways to modern purines and pyrimidines certainly support the proposal that Hadean conditions used a different route than those used now.

### 3.5. The Autocatalytic Origins of Proteins

**Figure 5:**
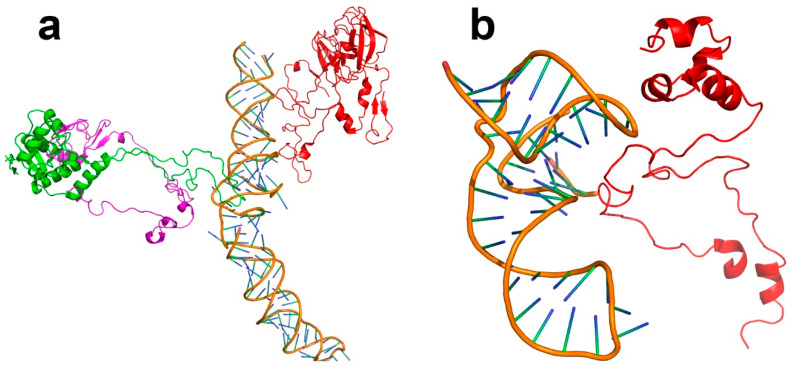
The original role of proteins was to stabilize RNA, rather than to act as enzymes. (**a**) The peptidyl transferase center of the *Escherichia coli* ribosome [96]. The figure shows the RNA that forms the catalytic site, as identified by [97], with the three proteins that form the closest association with it: the large subunit proteins L2 (red), L4 (green) and L15 (magenta). All of these have substantial non-globular parts, which are the parts in contact with the RNA. The catalytic center is in the middle of the RNA helix. (**b**) The human 17S U2 snRNP involved in the first step of RNA splicing [98], showing the RNA core and the protein most closely associated with it (splicing factor 3b). Again, the part of the protein in contact with the RNA is lacking in regular secondary structure.

Arguably the most primitive macromolecular catalysts still found in modern biology are the RNA catalysts. The best characterized of these is the ribosome. There are other examples of ribonucleoproteins, including snRNPs (involved in RNA splicing) and the signal recognition particle (SRP), which guides nascent proteins onto and through membranes. These all have an RNA function at their heart, with the protein forming a protective coat. This has been shown convincingly for the ribosome, of which the most primitive part is the peptidyl transfer center [93], which consists of a small piece of RNA, with a markedly non-globular protein stabilizing it (Figure 5a). Similar arrangements are found in snRNPs (Figure 5b) and the SRP [94]. It, therefore, seems likely (following Guideline 2) that the earliest proteins were simple polypeptides that stabilized RNA-like catalysts and later acquired catalytic functions as cofactors [95]. They would gradually acquire more defined sequences and functions—another example of autocatalytic selection.

## 4. Discussion

The guidelines set out here have provided guidance for how the major categories of modern metabolites came into being. The major additional step needed for life is the development of a genetic code and genes, on which there is a large literature [41,42,43,99]. There remain some significant gaps in our understanding, but the steps proposed here form a largely coherent pathway, with no *deus ex machina* required. The key innovation in these proposals is that prebiotic chemistry used not Darwinian natural selection but autocatalytic selection, which explains how the concentrations of pre-metabolites could have increased to a stage where the molecular pool became large enough to support metabolic processes. In each case, the original function of the metabolite was different from the one it has now, forcing us to consider new possibilities for prebiotic development. As set out here, these lead to a coherent and plausible route to life. It is worth noting that these proposals provide essentially all the metabolites needed for basic modern pathways, at reasonable concentrations. Thus, there is no fundamentally new chemistry required from here on.

**Figure 6:**
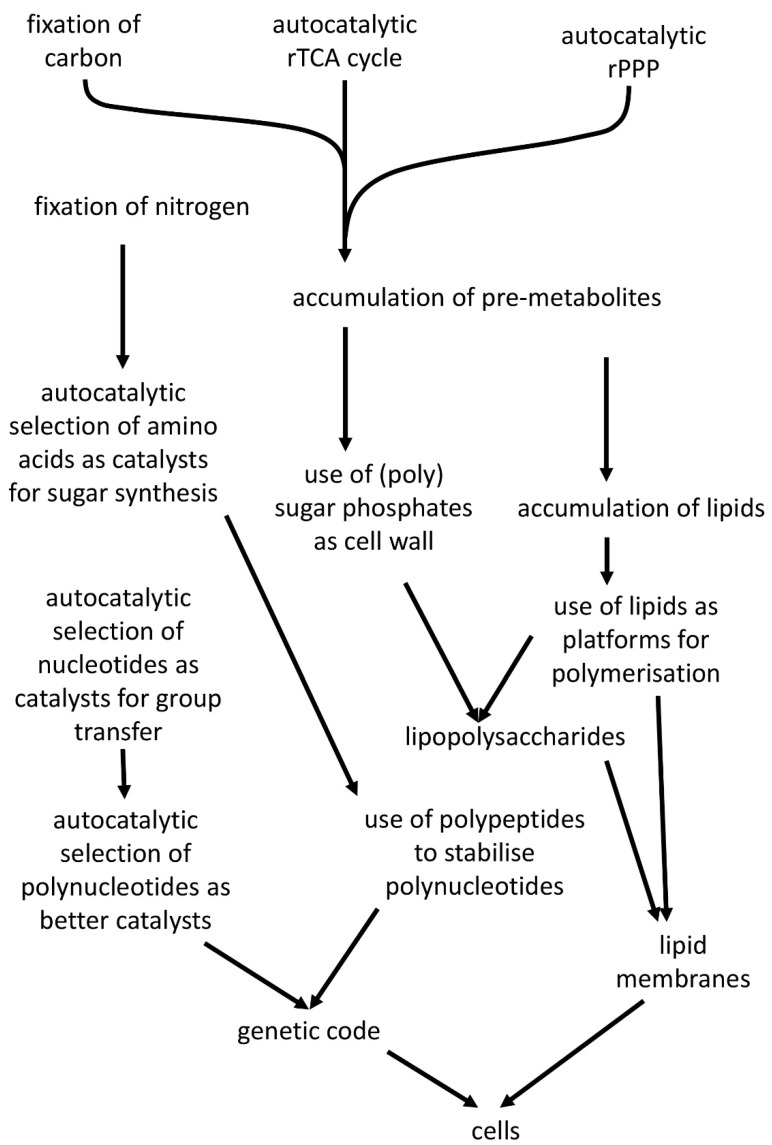
A possible timeline for the events described here. The diagram is not intended to present an indication of the length of time between events, but the events are intended to proceed in order. All of these processes require similar conditions such as a reducing environment, FeS surfaces, and a source of energy, most likely a geothermal vent. We suggest that currently the least obvious of these are the fixation of nitrogen and generation of nucleotide bases, both of which possibly arose from an abundant Hadean supply of hydrogen cyanide.

Historically, the biggest debate in prebiotic evolution has been over the question of which came first, genes or proteins. Our solution is that neither came first—sugar phosphates came first—and that for all the other major groups of modern metabolites, the original function was not the function that it now has, thus allowing autocatalytic selection to operate (Figure 6). This conclusion illustrates a key benefit of the guideline of autocatalytic selection—it forces us to see development not as a smooth linear route but as a series of opportunistic steps and then to go and identify those steps.

Francis Crick considered that the origin of life appeared “almost a miracle” [100]. If that is true, it implies that life in the universe may be extremely rare. On the other hand, if life is not rare, then the chances of our being able to identify recognizable life elsewhere are much greater. Many of the scenarios for the origins of various important molecules proposed to date have indeed been very unlikely and frequently rely on one unlikely set of conditions, followed by a second completely different and equally unlikely set. In this work, we set out to formulate proposals for and identify the most likely conditions for life. We will never know with confidence exactly what path life took. However, given that it took “only” a few hundred million years for life to arise on this planet, plausibility is an important criterion. The pathways proposed here are sufficiently plausible to be possible within this timescale. The concept of autocatalytic selection provides a mechanism for producing such plausible pathways. Our suggestions are in most cases supported by experimental evidence. There remain significant gaps, many of which are open to experimental investigation (Table 1).

We conclude that prebiosis requires simply a reducing atmosphere, a sustained energy source, such as a hydrothermal vent, and some common chemicals, such as water, hydrogen, CO, CO_2_, iron sulfide surfaces, and a source of nitrogen, but no obviously “special” conditions, with no dramatic changes in conditions required to progress all the way to life. It would therefore seem likely that life could have appeared on many other planets and that it could well have similar biochemistry to ours [10].

## Figures and Tables

**Table 1:** Key gaps in understanding that require experimental investigation.

Lipid vesicles able to catalyze (phospholipid) sugar polymerization?
Do phosphosugars form a barrier to diffusion of negatively charged pre-metabolites?
Polyphosphates as a route for producing sugar diphosphates.
Can HCN be produced in large amounts in Hadean conditions?
Are nitrogenous bases able to act as general nucleophilic catalysts?

## Data Availability

No new data were created in this study.
